# Supplementary material for: Identification and characterization of a human MORC2 DNA binding region that is required for gene silencing
Source: bioRxiv. 2024 Jun 6:2024.06.05.597643. Preprint. [Version 1] doi: 10.1101/2024.06.05.597643 (PMC11185635; doi:10.1101/2024.06.05.597643)
Supplement: Supplement 1 [file media-1.pdf]

SI Table 1. Identity of contaminant band (Fig1B) as assessed by mass spectrometry.

Species: *Trichoplusia ni*			
**Unique Counts**	**Total Counts**	**Protein Name**	**Gene ID**
89	95	Serine/threonine-protein kinase TOR	LOC113507625
83	91	acetyl-CoA carboxylase	LOC113498903
71	76	DmX-like protein 2 isoform X1	LOC113503468
68	74	Talin-1 isoform X4	LOC113499982
65	70	Triple functional domain protein isoform X1	LOC113504138
62	71	Dedicator of cytokinesis protein 7 isoform X3	LOC113501546
61	65	ubiquitinyl hydrolase 1	LOC113492721
59	63	U5 small nuclear ribonucleoprotein 200 kDa helicase	LOC113505650
55	62	Beta-galactosidase	LOC113504558
53	55	LOW QUALITY PROTEIN: nuclear pore complex protein Nup205	LOC113493667
47	52	Transcription elongation factor spt6	LOC113502227
44	48	CCR4-NOT transcription complex subunit 1 isoform X3	LOC113504324
41	43	Fatty acid synthase isoform X1	LOC113498344
39	43	Pre-mRNA-processing-splicing factor 8	LOC113502029
35	39	WD repeat and FYVE domain-containing protein 3 isoform X3	LOC113497332
34	36	Protein 4.1 homolog isoform X1	LOC113494423
34	36	Maternal protein tudor-like isoform X1	LOC113498000
34	36	Uncharacterized protein LOC113503664	LOC113503664
30	31	LOW QUALITY PROTEIN: baculoviral IAP repeat-containing protein 6-like	LOC113492500

Species: *Homo sapians*			
**Unique Counts**	**Total Counts**	**Protein Name**	**Gene ID**
95	305	ATPase MORC2	MORC2

SI Table 2. $K_{d,app}$ values for all proteins, substrates, and conditions tested.

**Protein**	**Substrate**	**Condition**	**$K_{d,app}$ (nM)**
Dephosphorylated MORC2	35mer random dsDNA	apo	17 ± 4
Phosphorylated MORC2	35mer random dsDNA	apo	285 ± 83
Phosphodead	35mer random dsDNA	apo	25 ± 3
Phosphomimetic	35mer random dsDNA	apo	512 ± 147
1-603	35mer random dsDNA	apo	446 ± 119
Aspartate mutant	35mer random dsDNA	apo	380± 120
Alanine mutant	35mer random dsDNA	apo	350± 60
Subset N	35mer random dsDNA	apo	90 ± 20
Subset C	35mer random dsDNA	apo	60 ± 20
Wildtype	35mer random dsDNA	apo	5 or less
Wildtype	149 bp Widom 601 dsDNA	apo	5 or less
Wildtype	Nucleosome	apo	16 ± 2
Wildtype	35mer ssDNA	apo	51 ± 9
Wildtype	35mer ssRNA	apo	49 ± 6
Wildtype	35mer high GC	apo	5 or less
Wildtype	35mer high AT	apo	5 or less
Wildtype	25mer random dsDNA	apo	21 ± 2
Wildtype	45mer random dsDNA	apo	11 ± 3
Wildtype	65mer random dsDNA	apo	5 or less
Wildtype	35mer random dsDNA	AMP-PNP	30 ± 5
Wildtype	35mer random dsDNA	ADP	36 ± 8
Wildtype	35mer random dsDNA	ATP	86 ± 15
Wildtype	35mer random dsDNA	ATPγS	275 ± 51

SI Table 3. Mechlorethamine and UV-induced protein-DNA crosslinking sites.

UV-induced crosslinking sites

index	RT	precursor m/z	score	charge	sequence	start	end	NuXL:NA	NuXL:NT	NuXL:best_localization	NuXL:best_localization_position	NuXL:best_localization_score	precursor_mz_error_ppm
10033	1512	836.351318	7.98E-06	2	AM(Oxidation)GEHLAQYWK	442	452	T	T	AMgEHLAQYWK	2	0.46096	4.82232659
21987	3187	1033.88635	0.003992589	2	DIQMAETSPEGTK	222	234	AG	A	DIQMAETSPEGTk	12	0.08188	-0.9964457
8647	1326	608.2481	0.003992664	4	DLDGM(Oxidation)FIYNCSRLIK	378	392	TT	T	DLDGMFIyNCSRLIK	7	0.67426	-3.6523695
12616	1858	823.322571	0.007701015	2	EDTM(Oxidation)TCLFLSR	122	132	A-H3N1	A	EDTMTCLfLSR	7	0.72102	-0.4642795
9392	1425	961.8713	7.62E-06	2	FDYVPTDTTPR	828	838	CT	C	FDYVpTDTTpR	9	0.05199	5.60342229
9267	1408	961.8706	0.003992478	2	FDYVPTDTTPR	828	838	CT	C	FDYVpTDTTpR	9	0.06368	4.87567012
8978	1369	817.347229	7.07E-06	2	FDYVPTDTTPR	828	838	T	T	FDyVPTDTTPR	2	1.01463	5.51259717
9834	1485	817.346619	7.06E-06	2	FDYVPTDTTPR	828	838	T	T	FDyVPTDTTPR	2	1.02506	4.76584609
9779	1477	817.347412	7.06E-06	2	FDYVPTDTTPR	828	838	T	T	FDyVPTDTTPR	2	1.04658	5.7366225
3540	659.8	757.31311	7.34E-06	2	FVVKEEK	764	770	AT	A	FVVKEEK	-1	0	4.31315599
4349	771.5	505.210999	8.26E-06	3	FVVKEEK	764	770	AT	A	FVVKEEK	-1	0	3.97936082
4226	754.7	765.311584	7.96E-06	2	FVVKEEK	764	770	GT	G	FVVKEEK	-1	0	5.59646638
4713	820.5	765.310852	8.25E-06	2	FVVKEEK	764	770	GT	G	FVVKEEK	-1	0	4.63943661
4085	735.1	600.781738	7.34E-06	2	FVVKEEK	764	770	T	T	FVVKEEK	-1	0	1.1614241
4806	833.2	752.807495	6.14E-06	2	FVVKEEK	764	770	TT	T	FVVKEEK	-1	0	4.56246897
4471	788.2	757.314087	7.10E-06	2	FVVKEEK	764	770	AT	A	FVVKeeK	5	0.00707	5.60267112
4570	801.5	757.313904	7.09E-06	2	FVVKEEK	764	770	AT	A	FVVKeeK	5	0.00941	5.36088703
4075	733.7	600.7842	7.40E-06	2	FVVKEEK	764	770	T	T	fVVKEEK	0	0.02402	5.25895479
4457	786.3	765.311218	7.40E-06	2	FVVKEEK	764	770	GT	G	FVVkEEk	6	0.02593	5.1179515

5093	871.7	752.808044	7.34E-06	2	FVVKEEK	764	770	TT	T	FvVKEEK	1	0.03597	5.2921627
4134	741.8	765.311157	0.003992613	2	FVVKEEK	764	770	GT	G	FVVkEEk	6	0.03685	5.03819901
3684	680	745.307678	7.34E-06	2	FVVKEEK	764	770	CT	C	FvVKEEK	1	0.07419	4.63001075
4054	730.8	745.307129	7.99E-06	2	FVVKEEK	764	770	CT	C	FvVKEEK	1	0.11419	3.89297427
3975	720.1	745.3083	6.70E-06	2	FVVKEEK	764	770	CT	C	FvVKEEK	1	0.18691	5.46427053
3903	710.2	745.307129	6.80E-06	2	FVVKEEK	764	770	CT	C	FvVKEEK	1	0.18979	3.89297427
3444	646.5	745.307678	7.34E-06	2	FVVKEEK	764	770	CT	C	FvVKEEK	1	0.33493	4.63001075
3378	637.2	745.307434	6.16E-06	2	FVVKEEK	764	770	CT	C	FvVKEEK	1	0.37333	4.30243898
4004	724.1	600.783936	4.45E-06	2	FVVKEEK	764	770	T	T	fVVKEEK	0	0.37715	4.81877258
3873	706	670.7843	0.003992545	2	GRFVVK	762	767	AT	A	GRFVVK	-1	0	4.83648033
4026	727.1	670.784058	7.30E-06	2	GRFVVK	762	767	AT	A	GRFVVK	-1	0	4.47513617
4184	748.8	670.784	7.91E-06	2	GRFVVK	762	767	AT	A	GRFVVK	-1	0	4.3892405
4250	757.9	670.7846	8.18E-06	2	GRFVVK	762	767	AT	A	GRFVVK	-1	0	5.28372015
4431	782.7	670.784546	7.30E-06	2	GRFVVK	762	767	AT	A	GRFVVK	-1	0	5.20306557
4519	794.7	670.783997	7.30E-06	2	GRFVVK	762	767	AT	A	GRFVVK	-1	0	4.384145
4582	803.1	670.783997	6.99E-06	2	GRFVVK	762	767	AT	A	GRFVVK	-1	0	4.384145
3728	685.9	658.777893	6.65E-06	2	GRFVVK	762	767	CT	C	GRFVVK	-1	0	3.72476817
4294	763.9	658.7786	8.18E-06	2	GRFVVK	762	767	CT	C	GRFVVK	-1	0	4.7978707
3942	715.5	678.781189	0.003992549	2	GRFVVK	762	767	GT	G	GRFVVK	-1	0	3.94191061
4077	734	678.780762	7.30E-06	2	GRFVVK	762	767	GT	G	GRFVVK	-1	0	3.31247688
4147	743.7	678.7816	7.61E-06	2	GRFVVK	762	767	GT	G	GRFVVK	-1	0	4.54746183
4210	752.5	678.781433	7.61E-06	2	GRFVVK	762	767	GT	G	GRFVVK	-1	0	4.30158703

4272	761	678.781433	0.003992545	2	GRFVVK	762	767	GT	G	GRFVVK	-1	0	4.30158703
4611	807	678.781189	8.20E-06	2	GRFVVK	762	767	GT	G	GRFVVK	-1	0	3.94191061
4674	815.3	678.782532	6.98E-06	2	GRFVVK	762	767	GT	G	GRFVVK	-1	0	5.92013091
4731	823	678.781494	7.30E-06	2	GRFVVK	762	767	GT	G	GRFVVK	-1	0	4.39150613
5126	875.9	678.781128	7.61E-06	2	GRFVVK	762	767	GT	G	GRFVVK	-1	0	3.8519915
5385	910.8	666.279053	7.91E-06	2	GRFVVK	762	767	TT	T	GRFvVK	3	0.00352	5.67398786
4127	740.8	658.778503	6.65E-06	2	GRFVVK	762	767	CT	C	gRFVVK	0	0.00612	4.65126227
4669	814.7	670.783691	7.88E-06	2	GRFVVK	762	767	AT	A	GRfVVK	2	0.01262	3.92918912
4614	807.4	666.27771	6.99E-06	2	GRFVVK	762	767	TT	T	GRFVvK	4	0.01607	3.65864451
3809	697.3	658.777832	8.18E-06	2	GRFVVK	762	767	CT	C	GRfVVK	2	0.01785	3.63211876
4064	732.2	658.77832	7.61E-06	2	GRFVVK	762	767	CT	C	gRFVVK	0	0.02852	4.37331404
4461	786.8	666.278625	7.30E-06	2	GRFVVK	762	767	TT	T	GRFVvK	4	0.05964	5.03274225
5299	899.2	666.27832	6.90E-06	2	GRFVVK	762	767	TT	T	GrFVVK	1	0.06294	4.57470967
4770	828.3	666.277893	6.33E-06	2	GRFVVK	762	767	TT	T	GRfVVK	2	0.15849	3.93346406
3544	660.4	576.251465	8.34E-06	3	GRFVVKEEK	762	770	AT	A	GRFVVKEEK	-1	0	2.80819964
3878	706.7	863.8746	7.81E-06	2	GRFVVKEEK	762	770	AT	A	GRFVVKEEK	-1	0	4.01484035
3278	623.3	851.869568	8.06E-06	2	GRFVVKEEK	762	770	CT	C	GRFVVKEEK	-1	0	4.75741264
3665	677.2	851.868652	8.35E-06	2	GRFVVKEEK	762	770	CT	C	GRFVVKEEK	-1	0	3.68268039
3457	648.2	707.3444	8.34E-06	2	GRFVVKEEK	762	770	T	T	GRFVVKEEK	-1	0	2.92890867
3814	697.9	576.2529	0.003992423	3	GRFVVKEEK	762	770	AT	A	GRFvvKEEK	4	0.00916	5.29871006
3456	648.1	851.869385	6.94E-06	2	GRFVVKEEK	762	770	CT	C	GRFVVkEEK	5	0.02006	4.54246619
3529	658.3	851.869751	6.23E-06	2	GRFVVKEEK	762	770	CT	C	GRfVVKEEK	2	0.03007	4.97235909

3904	710.4	871.872375	7.23E-06	2	GRFVVKEEK	762	770	GT	G	GRfVVKEEK	2	0.0409	4.34273353
4218	753.5	863.875366	0.003992421	2	GRFVVKEEK	762	770	AT	A	GRFvvKEEK	4	0.04651	4.90179086
3591	667	851.869507	8.09E-06	2	GRFVVKEEK	762	770	CT	C	GRFvvKEEK	4	0.05389	4.68576383
3631	672.5	863.8757	8.34E-06	2	GRFVVKEEK	762	770	AT	A	GRfVVKEEK	2	0.05545	5.28817842
4251	758	871.872253	7.23E-06	2	GRFVVKEEK	762	770	GT	G	GRFvvKEEK	4	0.06927	4.20272354
3844	702	851.8694	0.003992421	2	GRFVVKEEK	762	770	CT	C	GRFvvKEEK	4	0.07203	4.56034974
3828	699.9	871.87262	6.94E-06	2	GRFVVKEEK	762	770	GT	G	GRfVVKEEK	2	0.0751	4.62275351
3731	686.4	871.8737	7.47E-06	2	GRFVVKEEK	762	770	GT	G	GRfVVKEEK	2	0.08196	5.86189793
6219	1014	503.871521	0.003992573	3	IFIHGHK	255	261	GG-H2O1	G	IFiHGHK	2	0.03428	4.18750337
13548	1986	1051.8924	0.003992565	2	KEDTM(Oxidation)TCLFLSR	121	132	AG-H3N1	A	KEDtMtCLFLSR	5	0.11928	-4.0837475
4398	778.2	546.754211	0.003992627	2	KTESPIK	722	728	C-H3N1	C	KtESPIK	1	0.33112	3.52725315
14329	2093	844.824524	0.003992675	2	PSTEEPVRR	601	609	TA-H3N1	A	PsTEEPVRR	1	0.12161	-2.8379896
14402	2103	844.8244	0.00399267	2	PSTEEPVRR	601	609	TA-H3N1	A	PStEEPVRR	2	0.17279	-2.9846774
14991	2185	733.312317	0.00399262	2	QLTEKIR	563	569	CC-H2O1	C	QLTEkIR	4	0.06764	-4.2867793
7394	1163	662.9558	0.003992509	3	QQQEKLEALQK	570	580	AA	A	qqqEKLEALqK	9	0.176	-2.7374137
7455	1171	744.868958	0.007700944	2	QVQNRAITLR	334	343	C-H3N1	C	qVQNRAITLR	0	1.02484	-0.8301039
11829	1752	992.063904	0.007700994	3	QYEVGLQNLCNSYQSRADSR	953	972	AG-H3N1	A	QYEVgLQNLCNSYQSRADSR	4	0.15141	1.6019709
11479	1704	986.3979	7.58E-06	3	QYEVGLQNLCNSYQSRADSR	953	972	TT	T	QYEVGLQNLCNsYQsRADsR	18	0.1776	-0.5892887
11583	1718	986.731567	0.007700994	3	QYEVGLQNLCNSYQSRADSR	953	972	AA-H3N1	A	QYEVGLQNLcNSYQSRADSR	9	0.18494	0.90314665
11741	1740	992.062988	0.007700993	3	QYEVGLQNLCNSYQSRADSR	953	972	AG-H3N1	A	QYEVGlQNlCNSYQSRADSR	8	0.20737	0.67911824
11675	1731	986.398	0.007700993	3	QYEVGLQNLCNSYQSRADSR	953	972	TT	T	QYEVGLQNLCNsYQsRADsR	18	0.2855	-0.4879098
11401	1694	986.732727	7.34E-06	3	QYEVGLQNLCNSYQSRADSR	953	972	AA-H3N1	A	QYEVGLQnLCNSYQSRADSR	7	0.45275	2.07840957

11045	1647	992.062683	0.007700988	3	QYEVGLQNLCNSYQSRADSR	953	972	AG-H3N1	A	QYEVGlQNlCNSYQSRADSR	8	1.0785	0.37150069
10223	1537	785.872314	0.007701014	2	TLPFQLSSVEK	507	517	T	T	TlPFQlSSVEK	5	0.0569	-1.7108109
10347	1554	785.874084	6.14E-06	2	TLPFQLSSVEK	507	517	T	T	TLPfQLSSVEK	3	1.40683	0.54148435
12202	1802	724.9642	8.29E-06	3	VKFDYVPTDTTPR	826	838	TG-H3N1	G	VKFDYVpTDTTpR	11	0.22706	4.84000531
9274	1409	620.955017	8.03E-06	3	VKFDYVPTDTTPR	826	838	T	T	VKfDYVPTDTTPR	2	0.23548	4.80471219
10385	1559	930.929443	7.72E-06	2	VKFDYVPTDTTPR	826	838	T	T	VKFDyVPTDTTPR	4	0.89512	5.40452226
6571	1058	556.265686	7.99E-06	2	VPLGTFR	545	551	T	T	VpLGTFR	1	0.09573	5.15696366
7122	1128	720.791138	0.003992608	2	VPLGTFR	545	551	GT	G	vPLGTFR	0	0.10576	2.85707838
6480	1047	556.265381	7.09E-06	2	VPLGTFR	545	551	T	T	VPLgTFR	3	0.59214	4.60834568
6341	1029	556.264709	6.78E-06	2	VPLGTFR	545	551	T	T	VPLgTFR	3	2.40918	3.40138614

Mechlorethamine crosslinking sites

index	RT	precursor m/z	score	charge	sequence	start position	end position	NuXL:NA	NuXL:NT	NuXL:best_localization	NuXL:best_localization_position	NuXL:best_localization_score	precursor_mz_error_ppm
3472	637	756.7692	6.85E-06	2	CEASEQK	536	542	AT+C5H9N1	A	CeASEQK	1	0.1981	5.578125
4255	745	616.8087	5.68E-06	2	VTAVEVGK	812	819	G+C5H9N1	G	VTAVeVGK	4	0.8933	5.406465
4754	812	515.8949	7.03E-06	3	VTAVEVGK	812	819	AG+C5H9N1	A	VTAVeVGK	4	0.1817	5.84546
5112	861	510.5626	6.96E-06	3	VTAVEVGK	812	819	AA+C5H9N1	A	VTaVEVGK	2	0.0576	4.539196
5175	870	510.5628	0.0067337	3	VTAVEVGK	812	819	AA+C5H9N1	A	VTAVEVGK	-1	0	5.017378
5305	886	913.0333	6.32E-06	3	KDSNELSDSAGEEDSADLK	771	789	AA+C5H9N1	A	kDSNELSDSAGEEDSADLK	0	0.1898	2.902789
5553	920	760.8348	5.84E-06	2	VTAVEVGK	812	819	AT+C5H9N1	A	VTAVeVGK	4	0.9427	5.119838
5896	965	717.2875	0.0022799	2	TESPIK	723	728	GG+C5H9N1	G	TESpIK	3	0.0522	4.655995

6089	990	504.9027	5.54E-06	3	LSCCLYKPR	267	275	G+C5H9N1	G	LScCLYKPR	2	0.2037	5.580828
6178	1002	504.9029	6.24E-06	3	LSCCLYKPR	267	275	G+C5H9N1	G	LSCCLYkPR	6	0.0118	5.957218
6336	1023	504.9029	6.07E-06	3	LSCCLYKPR	267	275	G+C5H9N1	G	LSCCLYKPr	8	0.181	5.943485
6435	1036	504.9026	6.56E-06	3	LSCCLYKPR	267	275	G+C5H9N1	G	LsCCLYKPR	1	0.04	5.278614
6799	1084	669.2946	4.26E-06	3	SNAMAFTNYSSLNR	0*	11	G+C5H9N1	G	SNAmAFTNYSSLNR	3	1.6757	5.321795
7396	1163	1118.97	5.30E-06	2	SVAVSDEEEVEEEAER	739	754	G+C5H9N1	G	SvAvSDEEEvEEEAER	9	0.0075	3.685743
7847	1223	770.6443	4.66E-06	3	SNAMAFTNYSSLNR	0*	11	GT+C5H9N1	G	SNAmAFTNYSSLNR	3	1.2794	5.923861
9145	1391	858.6728	5.64E-06	3	DYPDTWVCSM(Oxidation)NPDPEQDR	518	535	C+C5H9N1	C	DYPDTWVCSMNPDPEQDr	17	0.7754	3.468842
9311	1412	861.0287	6.52E-06	3	QYEVGLQNLCNSYQSR	953	968	CC+C5H9N1	C	QYEVGlQNlCNSYQSR	8	0.4286	5.299045
10023	1508	960.0226	6.86E-06	3	DYPDTWVCSM(Oxidation)NPDPEQDR	518	535	CT+C5H9N1	C	DYpDTWVCSMNpDpEQDR	13	0.3493	4.251615
10152	1525	874.3621	5.40E-06	3	QYEVGLQNLCNSYQSR	953	968	CG+C5H9N1	C	QYEVGLQNLCNsYQSR	11	0.3193	2.905539
10233	1536	577.2558	6.58E-06	3	EDTMTCLFLSR	122	132	A+C5H9N1	A	EDTMTcLFLSR	5	0.357	5.242686
10315	1547	865.379	6.56E-06	2	EDTMTCLFLSR	122	132	A+C5H9N1	A	eDTMTCLFLSR	0	0.0914	3.983867
10317	1547	912.7424	6.95E-06	3	QYEVGLQNLCNSYQSRADSR	953	972	T+C5H9N1	T	QYeVGLQNLCNSYQSRADSR	2	0.2924	1.569192
10401	1558	865.3795	6.42E-06	2	EDTMTCLFLSR	122	132	A+C5H9N1	A	EDTMTCLFlSR	8	0.1782	4.548109
10551	1577	865.3782	6.79E-06	2	EDTMTCLFLSR	122	132	A+C5H9N1	A	EDtMtCLFLSR	4	0.1786	3.137504
10672	1593	576.2748	0.0067337	3	TNIVALLQK	999	1007	AA+C5H9N1	A	TNIVAllQK	6	0.8177	-5.322833
10726	1600	865.3782	6.38E-06	2	EDTMTCLFLSR	122	132	A+C5H9N1	A	EDTmTCLFLSR	3	0.1091	3.066974
108	1611	874.0329	3.82E-06	3	QYEVGLQNLCNSYQSR	953	968	AT+C5H9N1	A	QYEVgLQNLCNSYQSR	4	0.1185	5.868916

06													
10866	1619	865.3795	6.80E-06	2	EDTMTCLFLSR	122	132	A+C5H9N1	A	EDtMtCLFLSR	4	0.1221	4.548109
10933	1628	865.3786	6.73E-06	2	EDTMTCLFLSR	122	132	A+C5H9N1	A	EDtMtCLFLSR	4	0.033	3.559444
11856	1747	874.0305	4.13E-06	3	QYEVGLQNLCNSYQSR	953	968	AT+C5H9N1	A	QYEVGLQNLCnSYQSR	10	1.8209	3.123007
12573	1839	717.9372	0.0045219	3	DLDGMFIYNCSR	378	389	AT+C5H9N1	A	dLDGMFIYNCSR	0	0.6064	-4.403546
12741	1861	836.3563	6.97E-06	3	DLDGM(Oxidation)FIYNCSRLIK	378	392	AC+C5H9N1	A	DLDGMfIYNCSRLIK	5	0.8565	-4.160988
12758	1863	874.3637	0.0088962	3	QYEVGLQNLCNSYQSR	953	968	CG+C5H9N1	C	QYEVgLQNLCNSYQSR	4	0.1792	4.780573
13863	2010	572.2576	0.0067337	3	LLQPPEAPR	667	675	CT+C5H9N1	C	LLQppEApR	7	0.0258	-1.050692
14557	2101	1060.118	6.18E-06	3	EYFKQYEVGLQNLCNSYQSR	949	968	TT+C5H9N1	T	EYFKQYEVGLQNLCNsYQsR	18	0.0814	4.167658
14641	2113	726.2772	6.47E-06	3	DLDGM(Oxidation)FIYNCSR	378	389	AA+C5H9N1	A	DLDGMFIyNCSR	7	0.6885	1.888239
14646	2113	1060.118	6.29E-06	3	EYFKQYEVGLQNLCNSYQSR	949	968	TT+C5H9N1	T	EYFKQYEVGLQnLCnSYQSR	14	0.1186	4.450646
14996	2160	942.938	0.0045219	2	PANTLVKTASR	677	687	AA+C5H9N1	A	PANTlVKTASR	4	0.1258	3.044834
15086	2171	942.9363	0.0088962	2	PANTLVKTASR	677	687	AA+C5H9N1	A	PANTlVKTASR	4	0.1371	1.219997
15271	2196	942.9353	7.01E-06	2	PANTLVKTASR	677	687	AA+C5H9N1	A	PANTlVKTASR	4	0.1415	0.181435
15538	2231	700.8522	7.00E-06	2	EYRHLLR	435	441	A+C5H9N1	A	EYRHLlR	5	1.0685	1.777376

*The start position of this peptide includes a three amino acid scar from the cleavage of the affinity tag.

SI Table 4. V_{max} and $K_m^{app,ATP}$ of MORC2 in the presence of various DNA concentrations.

	**0μM DNA**	**0.05μM DNA**	**0.1μM DNA**	**1μM DNA**
V_{max} (min^{-1})	0.41 ± 0.02	0.36 ± 0.03	0.22 ± 0.02	0.17 ± 0.01
$K_m^{app, ATP}$ (μM)	0.4 ± 0.1	0.5 ± 0.1	0.4 ± 0.1	0.4 ± 0.1

SI Figure 1. Assessment of MORC2 protein and substrate purity.

- **A.** SDS-PAGE gel of purified MORC2 proteins stained with Coomassie blue stain. Gel was run with 10 µg of each protein to show the level of purity after size-exclusion chromatography.
- **B.** Circular dichroism spectrometry of wildtype and mutant MORC2. Spectra were taken at 25˚C (**Methods**).
- **C.** Native PAGE gel of reconstituted nucleosome stained with SYBR gold.

SI Figure 2. Representative spectra from mechlorethamine and UV-induced protein-DNA crosslinking mass spectrometry.

The relative intensity of MS/MS ions is plotted against their mass-to-charge ratios (*m/z*). Red corresponds to a and b ions, and green corresponds to y ions of the peptide. The sequence of the identified peptide is displayed with the detected ions colored in red or green. Spectra shown from **(A)** peptide fragment K722-K728 crosslinked by UV to dTMP, (**B**) peptide fragment G762-K767 crosslinked by UV to dCMP, (**C**) peptide fragment T723-K728 crosslinked by mechlorethamine to dGMP, and (**D**) peptide fragment S739-R754 crosslinked by mechlorethamine to dGMP. Spectra were exported from TOPPView (**Methods**) and for (**C**) the precursor ion ([M+2H] NM+GG^{2+}) was manually annotated. NM = nitrogen mustard or mechlorethamine.

SI Figure 3. Sequence alignment of MORC2 C-terminal domain.

MORC2 sequences from the indicated organisms were aligned in MAFFT and visualized in Jalview. Residues colored by percentage identity. Darker shades of blue indicate higher conservation. Phosphorylation sites are indicated in orange and putative DNA binding residues are indicated in pink.

SI Figure 4. ATPase activity and DNA binding of MORC2 mutants.

- **A.** Assessment of MORC2 ATPase activity. Phosphomimetic, subset N, subset C, alanine mutant, and phosphodead MORC2 (1 µM) were incubated with 1 mM ATP for 45 minutes at 37°C either in the presence or absence of 2 µM of a 35 base pair dsDNA. Inorganic phosphate released was quantified by malachite green (Methods). Error bars correspond to the standard deviation between three replicate experiments.
- **B.** Assessment of DNA binding by alanine mutant, subset N, and subset C MORC2. MORC2 constructs were titrated and incubated with 1 nM FAM-labelled of a 35 base

pair duplex DNA (Methods). Data were fit using a quadratic binding equation. Error bars correspond to the standard deviation between three replicate experiments.

C. MORC2 DNA binding to DNA sequences of different lengths as assessed by fluorescence anisotropy. MORC2 was titrated and incubated with 1 nM 5' FAM-labelled 25 base pair duplex DNA, 45 base pair duplex DNA, and 65 base pair duplex DNA (Methods). Data were fit using a quadratic binding equation. Error bars correspond to the standard deviation between three replicate experiments.

D. MORC2 DNA binding in the presence of ATP analogs. MORC2 was titrated and incubated with 1 nM 5' FAM-labelled 35 base pair duplex DNA in the presence or absence of 1 mM AMP-PNP, ATPγS, ADP, or ATP (**Methods**). Data were fit using a quadratic binding equation. Error bars correspond to the standard deviation between three replicate experiments.

SI Figure 5. Dimerization interfaces of MORC2.

AlphaFold Multimer model of full-length MORC2 as a dimer. Chain A is colored grey and chain B is colored by pLDDT score, as shown below the model. The end of the crystal structure model is indicated. Highlighted in magenta is the region of the C-terminus encompassing the phosphorylation sites and positively charged residues mutated.

SI Figure 6. Replicate gels from Figure 4.

A. N-terminal-maltose binding protein (MBP)-tagged wildtype, aspartate mutant, and E35A MORC2 (600 nM) were incubated with 100 nM supercoiled or linear pUC19. Samples were then added to amylose resin and washed in either low salt (50 mM NaCl) or high salt (400 mM NaCl) containing buffers before eluting the samples from the beads with maltose. Eluted samples were treated with proteinase K. DNA was resolved on a 1% (w/v) TAE agarose gel.

B. A biotin tagged DNA was conjugated to streptavidin magnetic beads to create a pseudo circular substrate. MORC2 (600 nM) was incubated with 20 µL of the beads in the presence or absence of 1 mM AMP-PNP. Supercoiled pBlueScript plasmid DNA (200 nM) was added before washing the beads with either low salt (50 mM NaCl) or high salt (400 mM NaCl) containing buffer. Samples were resuspended in 1X CutSmart buffer (New England Biolabs). DNA was released from the beads by digestion with ScaI and SbfI at 37°C for 1 hour before proteinase K treatment. DNA was resolved on a 1% (w/v) TAE agarose gel.

SI Figure 7. MORC2 localization analysis.

- **A.** NLS Stradmus analysis of potential nuclear localization sequences in MORC2 (**Methods**). Positively charged residues mutated in this study are shown below the graph in pink.
- **B.** Representative confocal microscopy images of interphase HeLa cells overexpressing EGFP- subset N, EGFP- E35A, EGFP- N39A, and EGFP- aspartate mutant MORC2.
- **C.** Representative confocal microscopy images of interphase HeLa cells overexpressing artificial NLS-EGFP, artificial NLS-EGFP- wildtype, and artificial NLS-EGFP- aspartate mutant MORC2.

SI Figure 8. MORC2 knockout validation.

- **A.** Sanger sequencing of MORC2 gene locus in parental and knockout HeLa cells.
- **B.** PCR amplification with primers designed to amplify a region of the MORC2 gene locus encompassing the deleted sequence of parental and knockout (KO) HeLa cell genomic DNA. DNA products were separated on a 1% TAE agarose gel and stained with SYBR safe. The PCR product of the native locus is 1199 bp.
- **C.** Western blot analysis of MORC2 protein level in parental and knockout (KO) HeLa cells. Beta-actin was used as a loading control.

SI Figure 9. RNA sequencing controls and analysis.

- **A.** Genome browser trace of MORC2 gene locus from NLS-EGFP (control), NLS-aspartate mutant, and NLS-wildtype MORC2 samples with exon 19, which contains the positive charge residue mutations in the aspartate mutant.
- **B.** Comparison of exogenous MORC2 RNA levels in NLS-EGFP (control), NLS-aspartate mutant, and NLS-wildtype MORC2 samples.
- **C.** Principle component analysis of the three biological replicates of NLS-EGFP, NLS-aspartate mutant, and NLS-wildtype MORC2 samples.
- **D.** Volcano plots of RNAseq reads for overexpression of wildtype MORC2 versus control, overexpression of aspartate mutant MORC2 versus control, and wildtype MORC2 versus aspartate mutant MORC2 without spike normalization (**Methods**). Significant upregulated genes are shown in green and significant downregulated

genes are shown in purple from three biological replicates. Significant genes are classified as those that meet the fold change > 1.5 and FDR > 0.05 cutoffs.

E. Venn diagram representation of the overlap between the significantly downregulated genes after overexpression of wildtype MORC2 identified in this study and previously identified targets of MORC2 silencing.

F. Representation of significant downregulated genes after overexpression of wildtype MORC2 in HeLa cells. 75 out of 197 genes are intronless or contain exons longer than 1 kb.

SI Fig 1

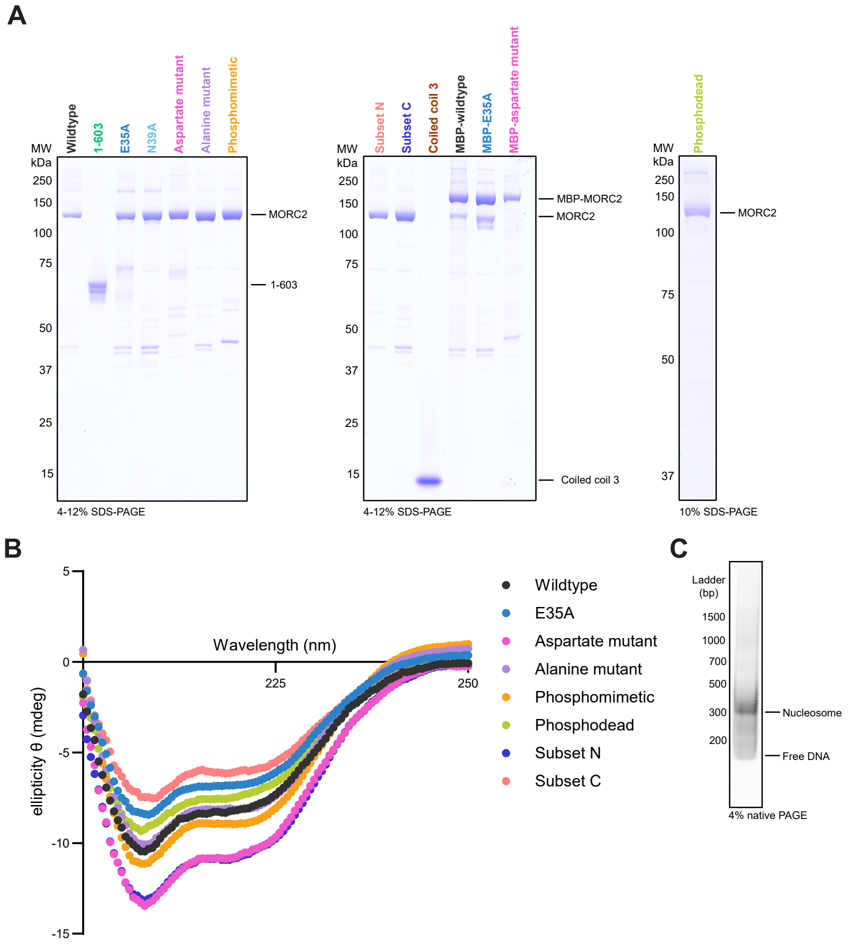
A
MW kDa
Wildtype
1-603
E35A
N39A
Aspartate mutant
Alanine mutant
Phosphomimetic
250
150
100
75
50
37
25
15
MORC2
1-603
4-12% SDS-PAGE
Subset N
Subset C
Coiled coil 3
MBP-wildtype
MBP-E35A
MBP-aspartate mutant
MBP-MORC2
MORC2
Coiled coil 3
4-12% SDS-PAGE
Phosphodead
MORC2
10% SDS-PAGE
B
Wavelength (nm)
225
250
ellipticity θ (mdeg)
5
0
-5
-10
-15
Wildtype
E35A
Aspartate mutant
Alanine mutant
Phosphomimetic
Phosphodead
Subset N
Subset C
C
Ladder (bp)
1500
1000
700
500
300
200
Nucleosome
Free DNA
4% native PAGE

SI Fig 2

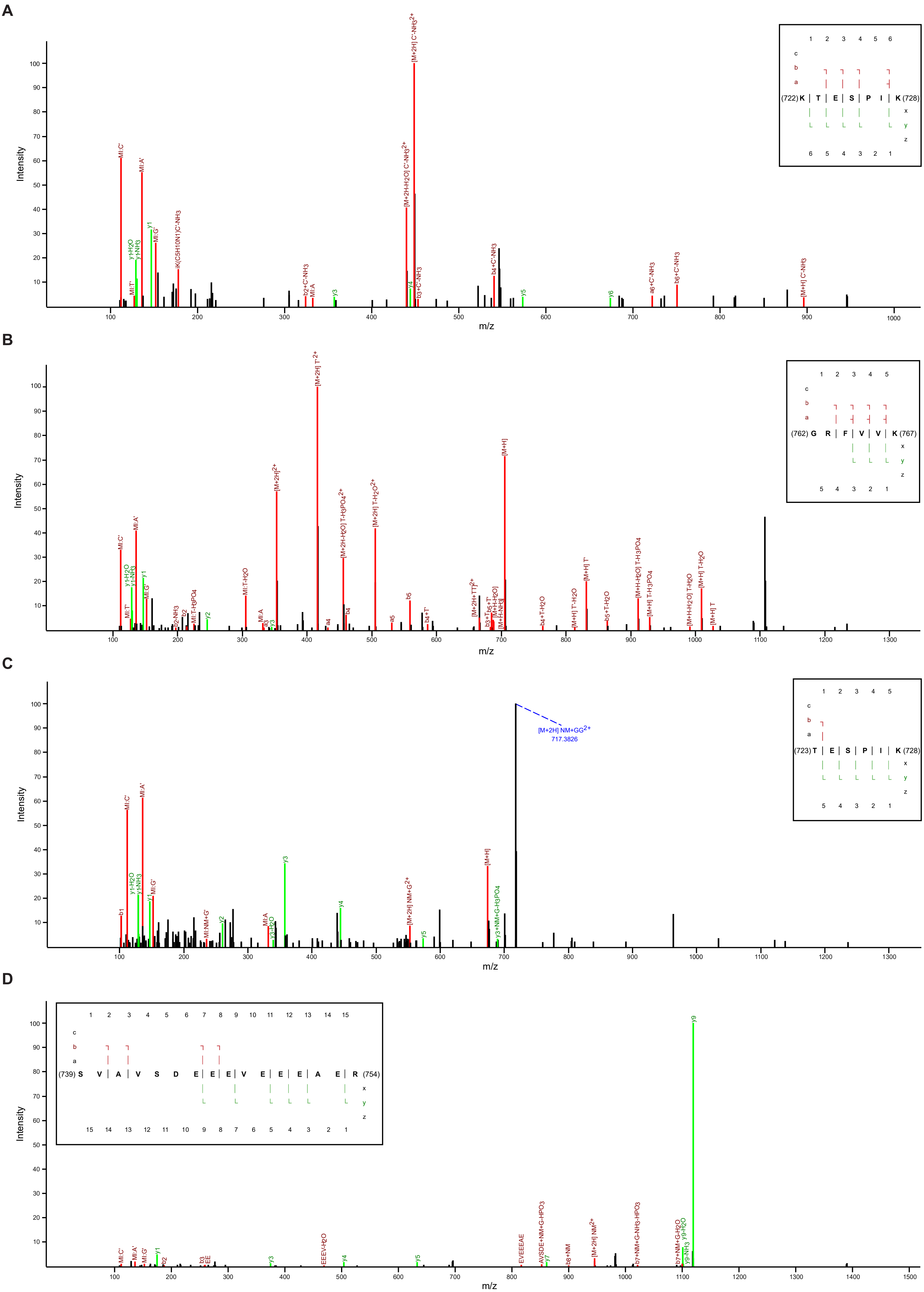
A
B
C
D
Intensity
m/z
(722) K T E S P I K (728)
(762) G R F V V K (767)
(723) T E S P I K (728)
(739) S V A V S D E E E V E E E A E R (754)
[M+2H] NM+GG2+
717.3826
[M+2H] C'-NH32+
[M+2H] T'2+
[M+H]
y9

SI Fig 3

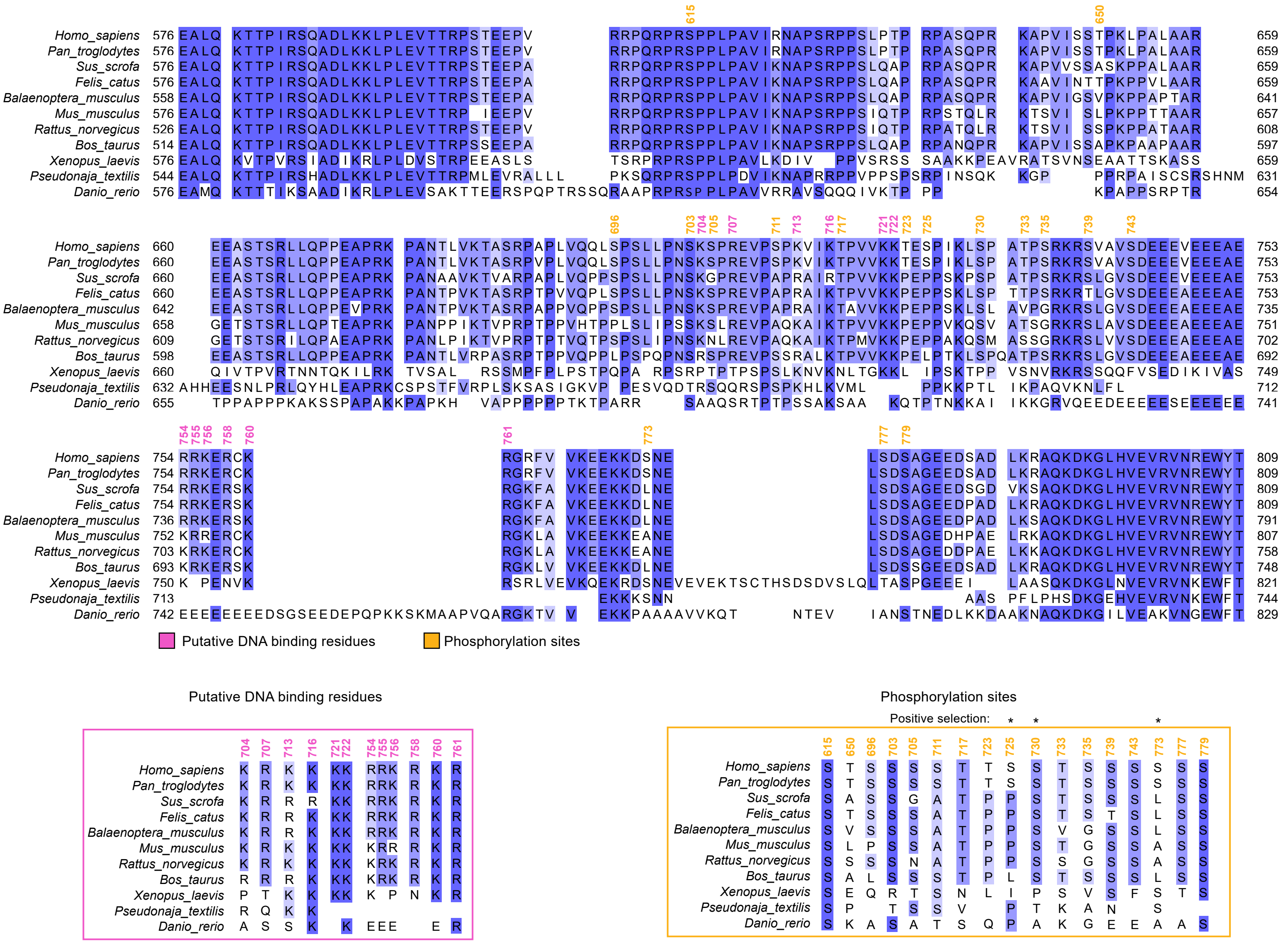

Homo_sapiens
Pan_troglodytes
Sus_scrofa
Felis_catus
Balaenoptera_musculus
Mus_musculus
Rattus_norvegicus
Bos_taurus
Xenopus_laevis
Pseudonaja_textilis
Danio_rerio
Putative DNA binding residues
Phosphorylation sites
Positive selection:

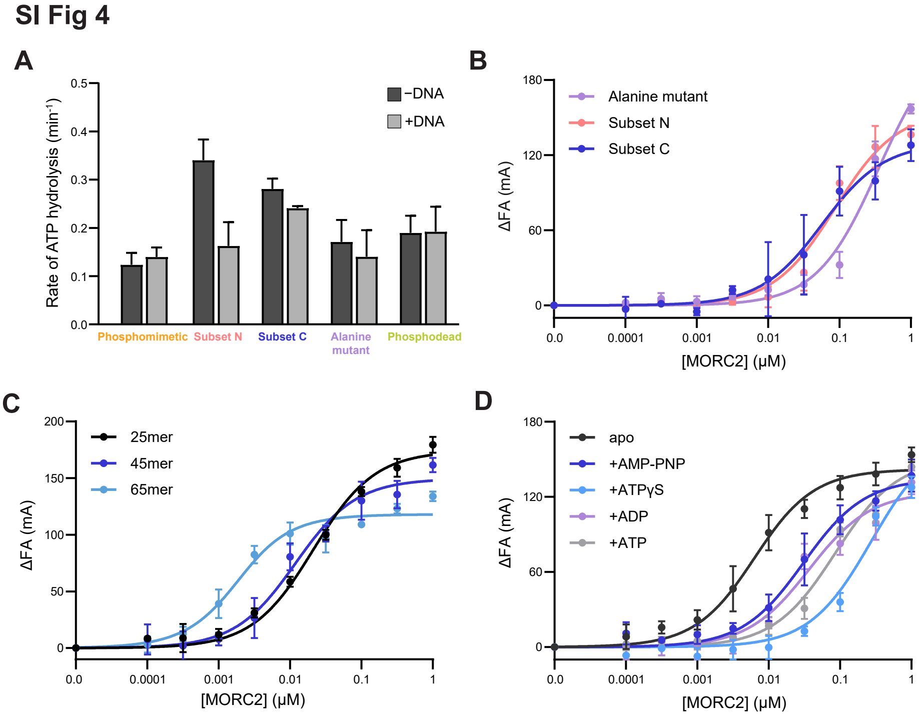
SI Fig 4
A
Rate of ATP hydrolysis (min⁻¹)
0.0
0.1
0.2
0.3
0.4
0.5
−DNA
+DNA
Phosphomimetic
Subset N
Subset C
Alanine mutant
Phosphodead
B
Alanine mutant
Subset N
Subset C
ΔFA (mA)
[MORC2] (μM)
C
25mer
45mer
65mer
ΔFA (mA)
[MORC2] (μM)
D
apo
+AMP-PNP
+ATPγS
+ADP
+ATP
ΔFA (mA)
[MORC2] (μM)
0.0
0.0001
0.001
0.01
0.1
1

SI Fig 5

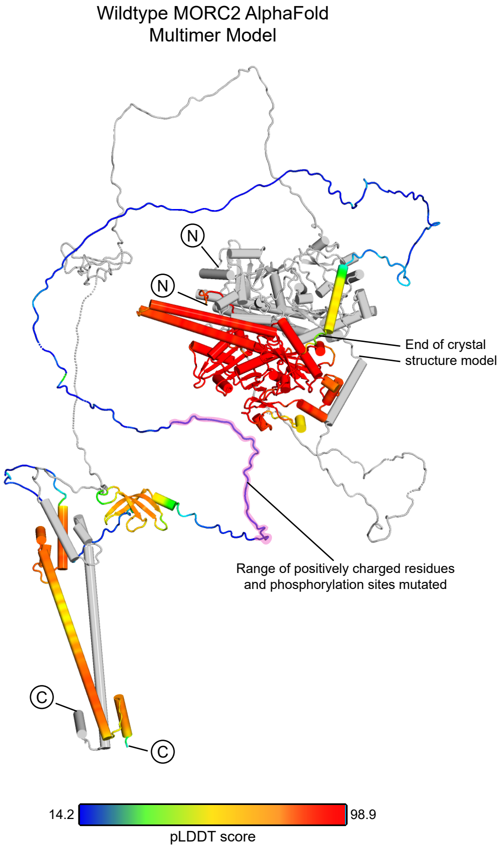
Wildtype MORC2 AlphaFold
Multimer Model
N
N
End of crystal
structure model
Range of positively charged residues
and phosphorylation sites mutated
C
C
14.2
98.9
pLDDT score

SI Fig 6

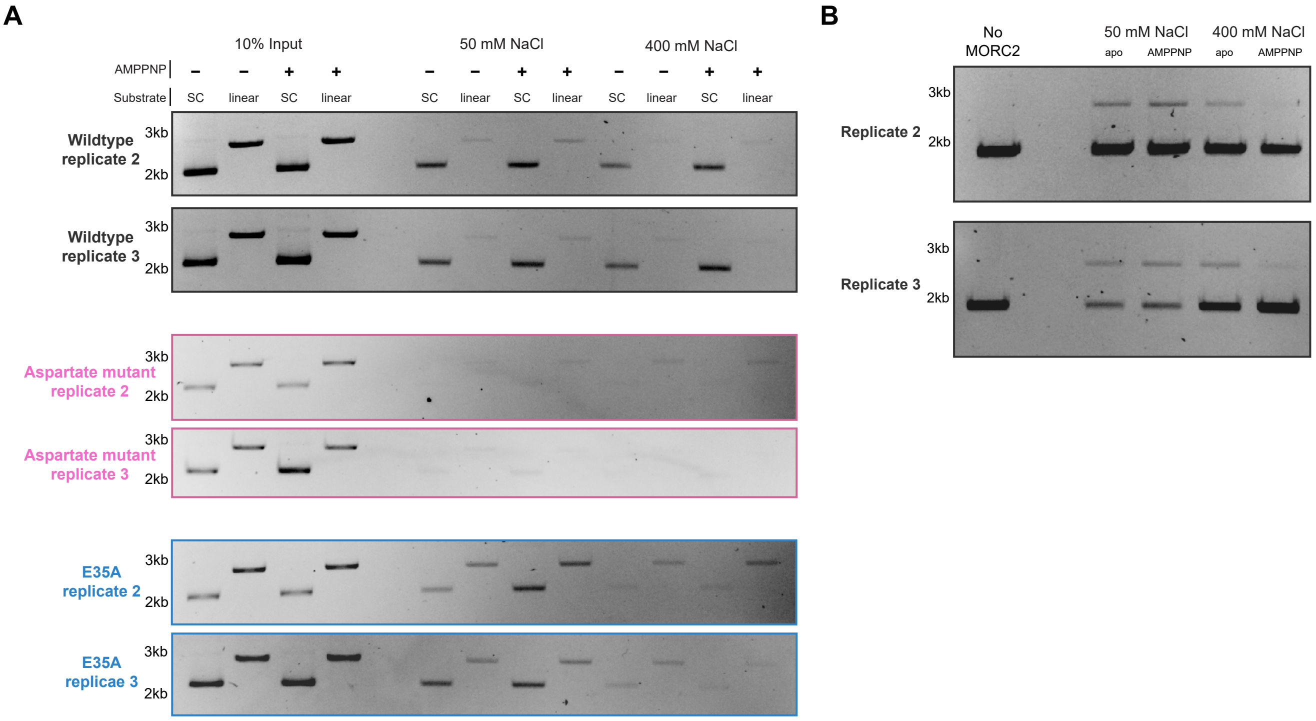
A
10% Input
50 mM NaCl
400 mM NaCl
AMPPNP
– – + + – – + + – – + +
Substrate
SC linear SC linear SC linear SC linear SC linear SC linear
Wildtype replicate 2
Wildtype replicate 3
Aspartate mutant replicate 2
Aspartate mutant replicate 3
E35A replicate 2
E35A replicae 3
3kb
2kb
B
No MORC2
50 mM NaCl
400 mM NaCl
apo AMPPNP apo AMPPNP
Replicate 2
Replicate 3
3kb
2kb

SI Fig 7

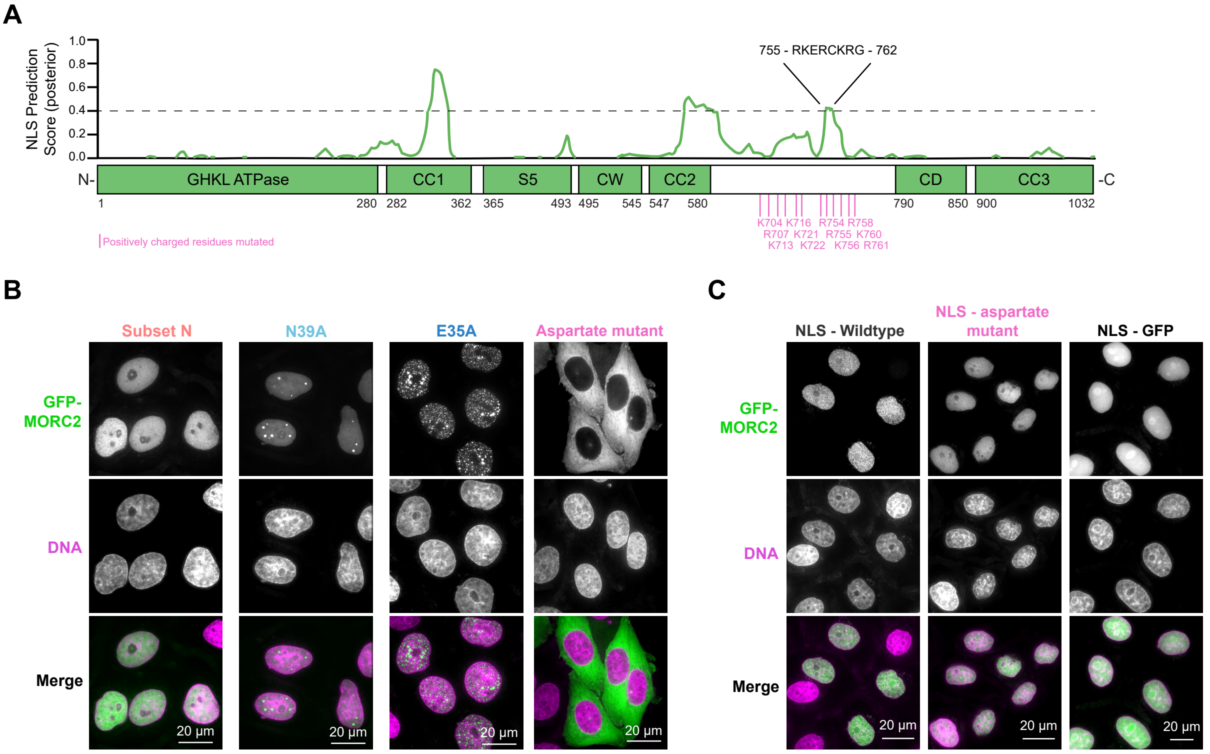
A
NLS Prediction Score (posterior)
1.0
0.8
0.6
0.4
0.2
0.0
755 - RKERCKRG - 762
N-
GHKL ATPase
CC1
S5
CW
CC2
CD
CC3
-C
1
280
282
362
365
493
495
545
547
580
790
850
900
1032
K704 K716 R754 R758
R707 K721 R755 K760
K713 K722 K756 R761
Positively charged residues mutated
B
Subset N
N39A
E35A
Aspartate mutant
GFP-MORC2
DNA
Merge
20 µm
C
NLS - Wildtype
NLS - aspartate mutant
NLS - GFP
GFP-MORC2
DNA
Merge
20 µm

SI Fig 8

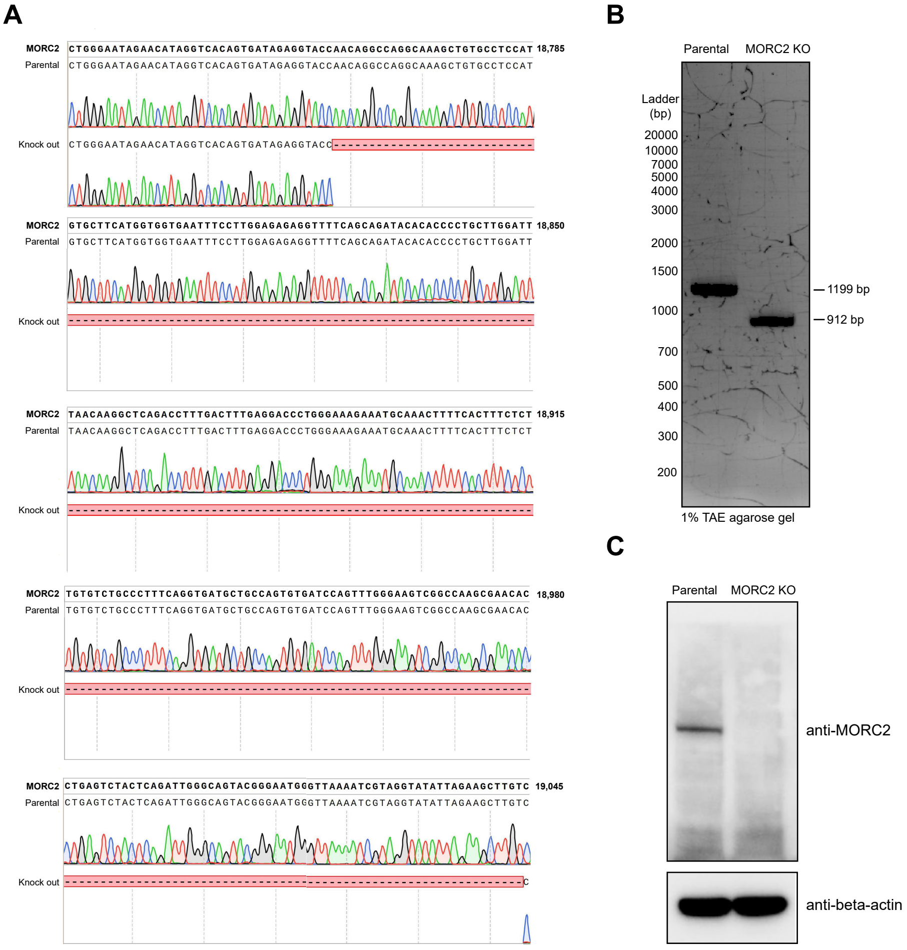
A
MORC2
CTGGGAATAGAACATAGGTCACAGTGATAGAGGTACCAACAGGCCAGGCAAAGCTGTGCCTCCAT
18,785
Parental
CTGGGAATAGAACATAGGTCACAGTGATAGAGGTACCAACAGGCCAGGCAAAGCTGTGCCTCCAT
Knock out
CTGGGAATAGAACATAGGTCACAGTGATAGAGGTACC
MORC2
GTGCTTCATGGTGGTGAATTTCCTTGGAGAGAGGTTTTCAGCAGATACACACCCCTGCTTGGATT
18,850
Parental
GTGCTTCATGGTGGTGAATTTCCTTGGAGAGAGGTTTTCAGCAGATACACACCCCTGCTTGGATT
Knock out
MORC2
TAACAAGGCTCAGACCTTTGACTTTGAGGACCCTGGGAAAGAAATGCAAACTTTTCACTTTCTCT
18,915
Parental
TAACAAGGCTCAGACCTTTGACTTTGAGGACCCTGGGAAAGAAATGCAAACTTTTCACTTTCTCT
Knock out
MORC2
TGTGTCTGCCCTTTCAGGTGATGCTGCCAGTGTGATCCAGTTTGGGAAGTCGGCCAAGCGAACAC
18,980
Parental
TGTGTCTGCCCTTTCAGGTGATGCTGCCAGTGTGATCCAGTTTGGGAAGTCGGCCAAGCGAACAC
Knock out
MORC2
CTGAGTCTACTCAGATTGGGCAGTACGGGAATGGGTTAAAATCGTAGGTATATTAGAAGCTTGTC
19,045
Parental
CTGAGTCTACTCAGATTGGGCAGTACGGGAATGGGTTAAAATCGTAGGTATATTAGAAGCTTGTC
Knock out
C
B
Parental
MORC2 KO
Ladder (bp)
20000
10000
7000
5000
4000
3000
2000
1500
1000
700
500
400
300
200
1199 bp
912 bp
1% TAE agarose gel
C
Parental
MORC2 KO
anti-MORC2
anti-beta-actin

SI Fig 9

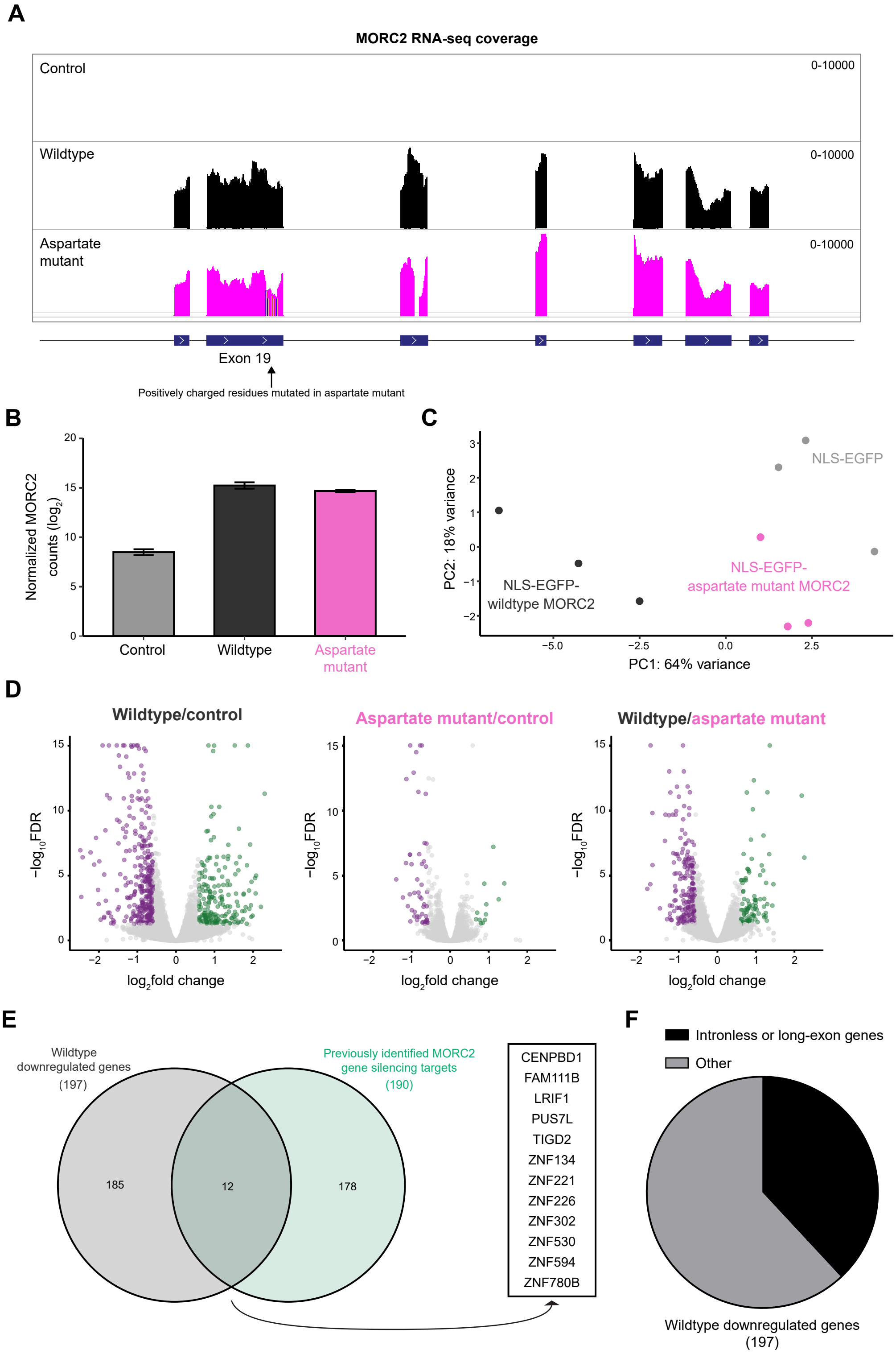
A
MORC2 RNA-seq coverage
Control
0-10000
Wildtype
0-10000
Aspartate mutant
0-10000
Exon 19
Positively charged residues mutated in aspartate mutant
B
Normalized MORC2 counts (log_2)
20
15
10
5
0
Control
Wildtype
Aspartate mutant
C
PC2: 18% variance
PC1: 64% variance
NLS-EGFP
NLS-EGFP-wildtype MORC2
NLS-EGFP-aspartate mutant MORC2
−5.0
−2.5
0.0
2.5
D
Wildtype/control
Aspartate mutant/control
Wildtype/aspartate mutant
$-log_{10}FDR$
log_2fold change
E
Wildtype downregulated genes (197)
Previously identified MORC2 gene silencing targets (190)
185
12
178
CENPBD1
FAM111B
LRIF1
PUS7L
TIGD2
ZNF134
ZNF221
ZNF226
ZNF302
ZNF530
ZNF594
ZNF780B
F
Intronless or long-exon genes
Other
Wildtype downregulated genes (197)
